# Metal distribution in three organs and edibility assessment on* Coptodon rendalli* from the Umgeni River impacted by metallurgic industrial activities

**DOI:** 10.1007/s10661-024-12875-w

**Published:** 2024-07-17

**Authors:** Sanelisiwe Siphumelele Brightness Hlatshwayo, Ajay Bissessur, Mapurunyane Callies Selala, Yuki Takai, Jeffrey Lebepe

**Affiliations:** 1 School of Life Sciences, University of KwaZulu-Natal: Westville Campus, Durban, South Africa; 2 School of Chemistry and Physics, University of KwaZulu-Natal: Westville Campus, Durban, South Africa; 3 Department of Biology and Environmental Sciences, School of Science and Technology, Sefako Makgatho Health Science University, Pretoria, South Africa; 4 Laboratory of Marine Environmental Science, Faculty of Agriculture, Kyushu University, Fukuoka, Japan

**Keywords:** Liver, Gill, Muscle, Metal pollution, Bioaccumulation, Antimony, Chromium, Lead

## Abstract

Fish is among the most affordable and readily available protein sources for communities residing near water bodies. However, the recent pollution status of aquatic ecosystems has rendered fish consumption risky for human health. The study evaluated metal levels in the liver, gill, and muscle tissues of Redbreast tilapia (*Coptodon rendalli*) from Inanda and Nagle dams in the uMgeni River system. Metals, Al, Sb, Cd, Cr, Fe, Mn, Mo, Pb, and Zn were analysed using ICP-OES. Fish size showed no significant difference between the two dams (p > 0.05) whereas a descending trend liver > gill > muscle was observed for most metal levels at both dams. Moreover, there was a clear separation for metal levels in the liver, gill, and muscle between the two dams (p < 0.001) and a similar trend was observed for organs in each dam (p < 0.001). No relationship was observed between fish length and metal levels and no definite trend was observed for inter-metal relationships. Antimony, Cr, and Pb showed THQs greater than 1 at both dams which suggests health risks for consumers. Molybdenum has also shown a concerning THQs with some individuals exhibiting values ranging from 0.5 – 0.9. These findings suggest that consuming *C. rendalli* from the Inanda and Nagle dams could result in adverse health effects from Sb, Cr and Pb.

**Supplementary Information:**

The online version contains supplementary material available at 10.1007/s10661-024-12875-w.

## Introduction

Fish are important components of the aquatic food chain as they occupy higher trophic levels, hence, they can be used as accumulative indicators (Ali et al., 2020a, 2020b; Peel et al., 2019; Vander Zanden & Vadeboncoeur, 2002). According to Rajeshkumar and Li (2018), fish can accumulate higher metal levels than water and sediment. Organs such as the liver and gills can accumulate elevated metal levels due to their role in metabolism whereas muscle accumulates less. The liver is known for biotransformation and detoxification making it a target organ for chemicals whereas gills are located externally and play a role in gaseous exchange and osmoregulation (Cui et al., 2020; Roberts, 2012; Wood & Eom, 2021). Despite accumulating lesser levels, muscle tissue inclusion is crucial in bioaccumulation studies as it is the part consumed by humans.

Fish forms not only an integral part of aquatic ecosystems but is an important food source for humans around the world, especially in rural areas dominated by unemployed individuals. Fish has a high content of proteins, essential fats, vitamins, and minerals (Esilaba et al., 2020; Pan et al., 2019). Moreover, Addo-Bediako et al. (2014) reported that fish are cheap protein sources that are easily accessible from local aquatic systems such as rivers and dams. Consuming fish is beneficial for human health as it reduces the risks of coronary heart disease, and hypertension and prevents certain cardiac arrhythmias (Pan et al., 2019; Storelli, 2008).

However, aquatic ecosystems have now become polluted resulting in uncertainties regarding the safety of consuming their inhabitant fish. According to Ali et al. (2023), fish consumption has become one of the routes of element exposure for humans. Studies showed that frequent consumption of fish from polluted water bodies may pose risks to human health (Adegbola et al., 2021; Hao et al., 2022). Therefore, frequent monitoring of the level of contaminants in fish that are consumed by humans for their livelihood should be the order of the day. Moreover, it is imperative to understand the distribution of these elements in fish organs, the relationship between fish size and element content, the inter-metal relationships within each tissue, as well as their extent of bioaccumulation in portions consumed by humans, particularly in water bodies where subsistence fishing is commonplace.

The uMgeni River is among the most polluted systems in the country and the catchment is dominated by rural poor communities which opt for fish as the protein source. However, the health risks associated with the consumption of these fish are scantly reported. Tilapia species are the most preferred by these communities with *Coptodon rendalli* showing to be a commonly occurring and the most abundant at both dams. Therefore, the present study aims to assess element levels in the liver, gill, and muscle, and the risks associated with consuming *Coptodon rendalli* from the uMgeni River system. The Nagle Dam is located upstream whereas Inanda Dam is located downstream of the uMgeni-uMsunduzi rivers confluence. The uMsunduzi River drains a highly industrialized catchment and has been reported to be one of the most polluted rivers in South Africa (Ngubane et al., 2023; Olaniran et al., 2014). It was, therefore, hypothesised that the Inanda Dam populations will exhibit relatively higher metal levels compared to those from Nagle Dam and fish from the former will not be safe for human consumption.

## Materials and methods

### Study sites

**Fig. 1:**
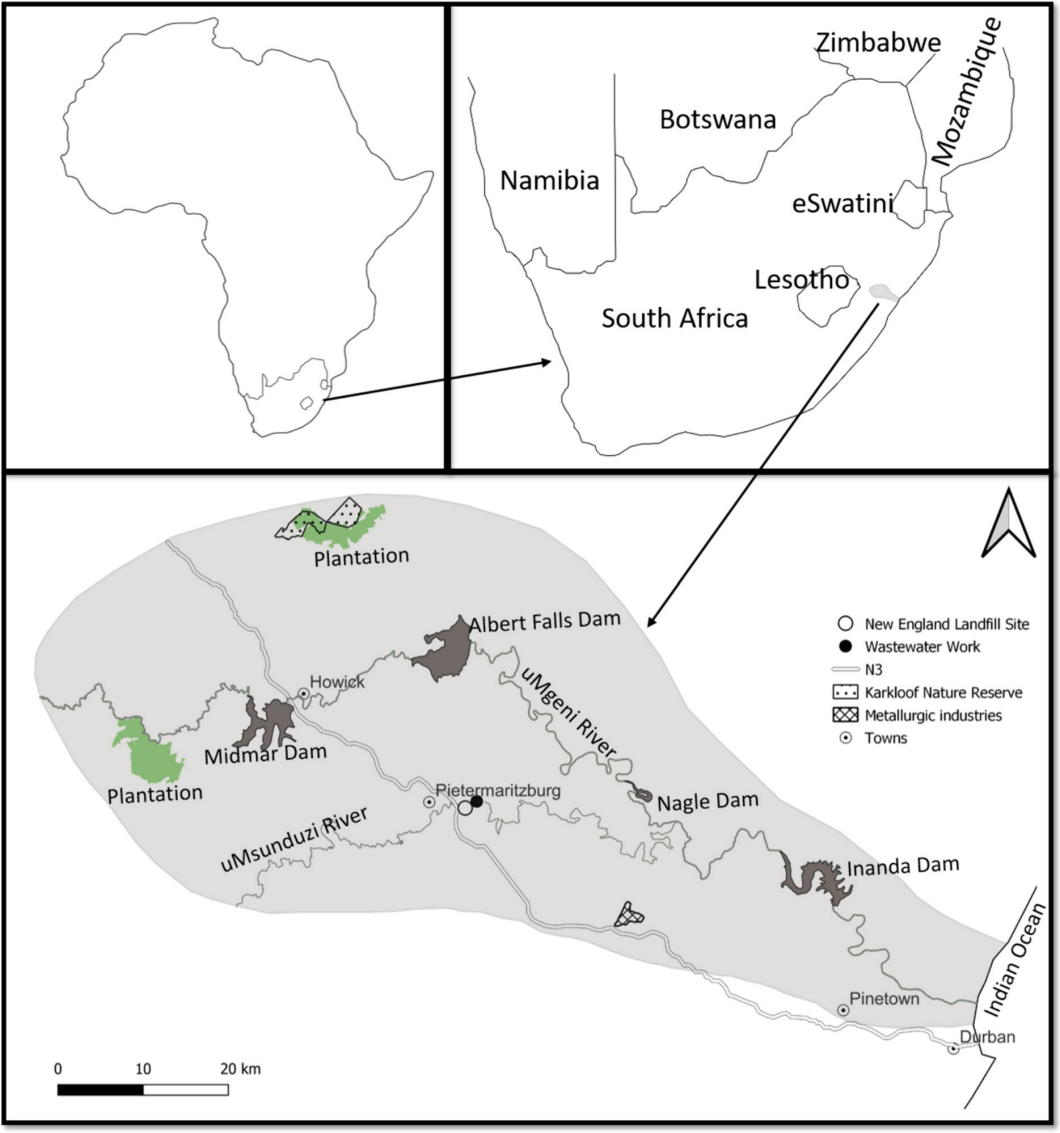
The uMgeni River catchment showing all four impoundments along the system with study area being the two in the lower stretch

The Nagle and Inanda dams are located in the lower reach of the uMgeni River system which feeds the Indian Ocean in Durban in South Africa (Fig. 1). The Nagle Dam is bordered by mountains and rural areas and is the first impoundment of the lower uMgeni River stretch. As the flows downstream, it is joined by the polluted uMsunduzi River before feeding the Inanda Dam. The uMsunduzi River drains approximately 540 km^2^ catchment dominated by metallurgic industries, hence, it is described as the main driver of metal pollution in the Inanda Dam (Misra et al., 2024; Olaniran et al., 2014).

### Fish sampling and processing

A total of 43 fish specimens of *C. rendalli* were collected from Inanda (n = 31) and Nagle (n = 12) dams during low flow (Aug—Oct 2020) and high flow (Dec 2020—Jan 2021) surveys. Fish were collected with the aid of an electro-shocker and gill nets with mesh sizes ranging from 35 to 90 mm stretch. Fish identification was carried out using Skelton (2001). Live fish were stored in tanks filled with dam water and aerated to increase the oxygen level and minimize stress. Fish were euthanised and dissected ventrally to harvest internal organs such as the liver and gills. A measuring board was used to measure fish lengths (cm) with a digital spring scale used for fish weight. The spring scale had a sensitivity of 10 g. After recording the weight and length, its gender was recorded by observing the gonad tissue. A sample of gill, liver, and muscle tissues was harvested from each fish specimen and washed with distilled water. The liver was weighed using a KERN analytical scale with a sensitivity of 0.001 g (KernADB 210 g:0.0001 g). Each sample was wrapped with aluminium foil and kept on ice at the site and later transferred to -80℃ ultra freezer until elements analysed. The study was approved by the Animal Research Ethics Committee of the University of KwaZulu-Natal (Ref: AREC/019/018).

### Overall fish condition

The overall fish condition was determined using a length–weight relationship, condition factor (CF), and hepatosomatic index (HSI). The fish length–weight relationship was calculated following the Jisr et al. (2018) protocol where a linear regression of the log-transformed equation was used (Eq. 1). Fish length and weight were used to calculate the condition factor as per Bagenal and Tesch (1978) protocol using Eq. 2. Bagenal and Tesch (1978) documented that the condition factor < 2 denotes poor health condition whereas condition factors ranging from 2.9 to 4.8 signify healthy fish condition. The hepatosomatic index was calculated following Gupta et al. (2017) protocol using Eq. 3.

1 $$\text{log}\left(\text{W}\right)=\text{log }\left(\text{a}\right)+\text{b log }\left(\text{L}\right)$$

where ‘a’ represents the intercept and ‘b’ is the slope of the relationship. The parameter ‘b’ could either be 3, > 3, or < 3. The ‘b’ of 3 denotes isometric growth, and b > 3 represents positive allometric growth which denotes the optimum condition for growth, whereas b < 3 represents negative allometric growth where fish become slimmer as they grow (Jisr et al., 2018).

2 $$\text{CF}=\frac{\text{W x }10}{{\text{L}}^{3}}$$

where CF is the condition factor, W is the fish weight (g) and L is the total fish length in (cm)

3 $$\text{HSI}=\frac{\text{Liver weight }(\text{g})}{\text{Body weight }(\text{g})}\text{ x }100$$

Laboratory analyses.

In the laboratory, tissue samples were thawed and dried in an oven at 110 $^\circ{\rm C}$ for 48 h. The digestion of each sample (0.2 g) was performed on a hotplate using a prepared aqua regia mixture (3HCl:1HNO_3_) (Muinde et al., 2013). Analytical grade chemicals were used for all sample digestions. The solutions were double filtered through a 0.45 µm filter paper and 0.45 µm syringe filter and then made up to 250 ml with deionised water. Inductively Coupled Plasma Optical Emission Spectrometry (ICP-OES) was used for all metal analyses. The analyses were carried out with blanks and certified reference materials (CRMs) and the recovery for the CRMs ranged from 86 – 102%.

### Human health risk assessment

A protocol by US Environmental Protection Agency US-EPA (2000) was used to assess the edibility of fish by calculating target hazard quotient (THQ) for non-carcinogenic risk. The THQ for each metal was calculated using the Eq. 4.

4 $$\text{THQ }= \frac{\text{C }\times \text{ EF }\times \text{ ED }\times \text{ IRF }}{\text{RfD }\times \text{ BW X AT}}$$

where C is the concentration in muscle, EF is the exposure frequency, IRF is the ingestion rate, RfD is the reference dose, Bw is the body weight, and AT is the average time. The THQ < 1 entails the unlikeliness of adverse health effects whereas the THQ > 1 suggests possible adverse health effects (US-EPA, 2000). The RfD by USEPA (2013) and Ashraf et al. (2012) were used for calculating THQs. Target hazard quotients of metal were summed to give Hazard Index (HI) using Eq. 5.

5 $$\text{HI}- {\text{THQ}}_{\text{A}1}+ {\text{THQ}}_{\text{Sb}}+ {\text{HQ}}_{\text{Cd}}+ {\text{HQ}}_{\text{Fe}}+ {\text{THQ}}_{\text{Fe}}+ {\text{THQ}}_{\text{Mn}}+ \dots \dots + {\text{THQ}}_{\text{n}}\dots \dots$$

Box-and-whisker plots were prepared for the THQ values derived from the human health risk assessment for both the Inanda and Nagle dams using R-4.3.1. Moreover, the carcinogenic risks were assessed using the cancer slope factors provided by USEPA (2010), following Eq. 6 as per Adegbola et al. (2021). The slope factors available were for Cd, Cr, and Pb, and the acceptable limit for lifetime exposure ranges from 10^–6^ – 10^–4^ (Oni et al., 2022).

6 $$\text{THQ }= \frac{\text{C }\times \text{ EF }\times \text{ ED }\times \text{ IRF }\times \text{CSF }}{\text{RfD }\times \text{ BW X AT}}$$

With CSF being the cancer slope factor.

### Data analysis

Levene’s and Shapiro–Wilk's tests were used to test the assumptions of homogeneity of variance and normality of the data, and the assumptions were not satisfied (p < 0.05). Therefore, the Wilcoxon-Mann–Whitney U-test was used to compare metal concentrations between the two dams. Non-metric multidimensional scaling was used to check the metal trends across the three tissues whereas the relationship was evaluated using Spearman’s correlation test. The R software (R-4.3.1) was used for all statistical analyses with a significance set at p < 0.05.

## Results

### Overall fish health

The size of *C. rendalli* was fairly similar between the two dams and the length–weight relationship has also shown negative allometric growth for both Inanda and Nagle Dam populations (Table 1). The condition factor ranged from 1.53 – 3.86 (x̄ = 2.69 ± 0.74) at Nagle Dam and 1.43 – 4.11 (x̄ = 2.64 ± 0.69) at Inanda Dam. No significant difference was recorded for the condition factor between the two populations (p > 0.05). Similarly, the hepatosomatic index (HSI) exhibited no significant difference between the two populations (p > 0.05) with values ranging from 0.51 – 3.23% (x̄ = 1.09 ± 0.73) being recorded at Nagle Dam and 0.52 – 2.19% (x̄ = 1.18 ± 0.41) at Inanda Dam.

**Table 1:** Length–weight relationship of *Coptodon rendalli* observed at Inanda and Nagle dams

Dams	Sex	n	b	a	R ^2^	Growth
Inanda	Males	14	2.066	0.00687	0.885	-Allometric
	Females	17	1.255	0.54413	0.839	-Allometric
	Combined	31	1.619	0.07860	0.723	-Allometric
Nagle	Males	4	1.352	0.32018	0.884	-Allometric
	Females	8	1.174	0.03120	0.852	-Allometric
	Combined	12	1.622	0.06931	0.887	-Allometric

### Metal accumulation

**Fig. 2:**
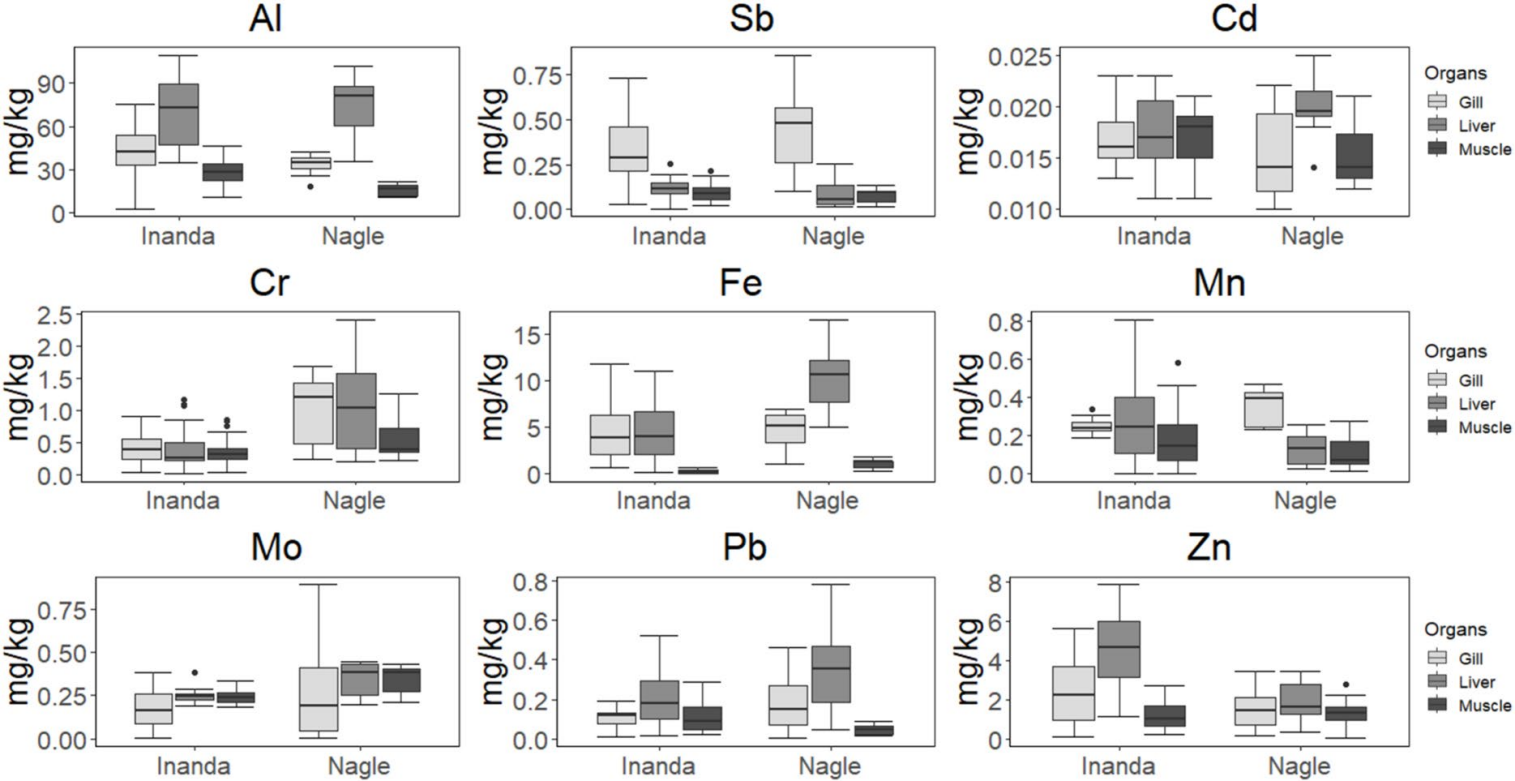
Box and whisker plots display the minimum, first quartile, median, third quartile, and maximum value in each sample (outliers are represented as dots) from Inanda and Nagle dams

**Fig. 3:**
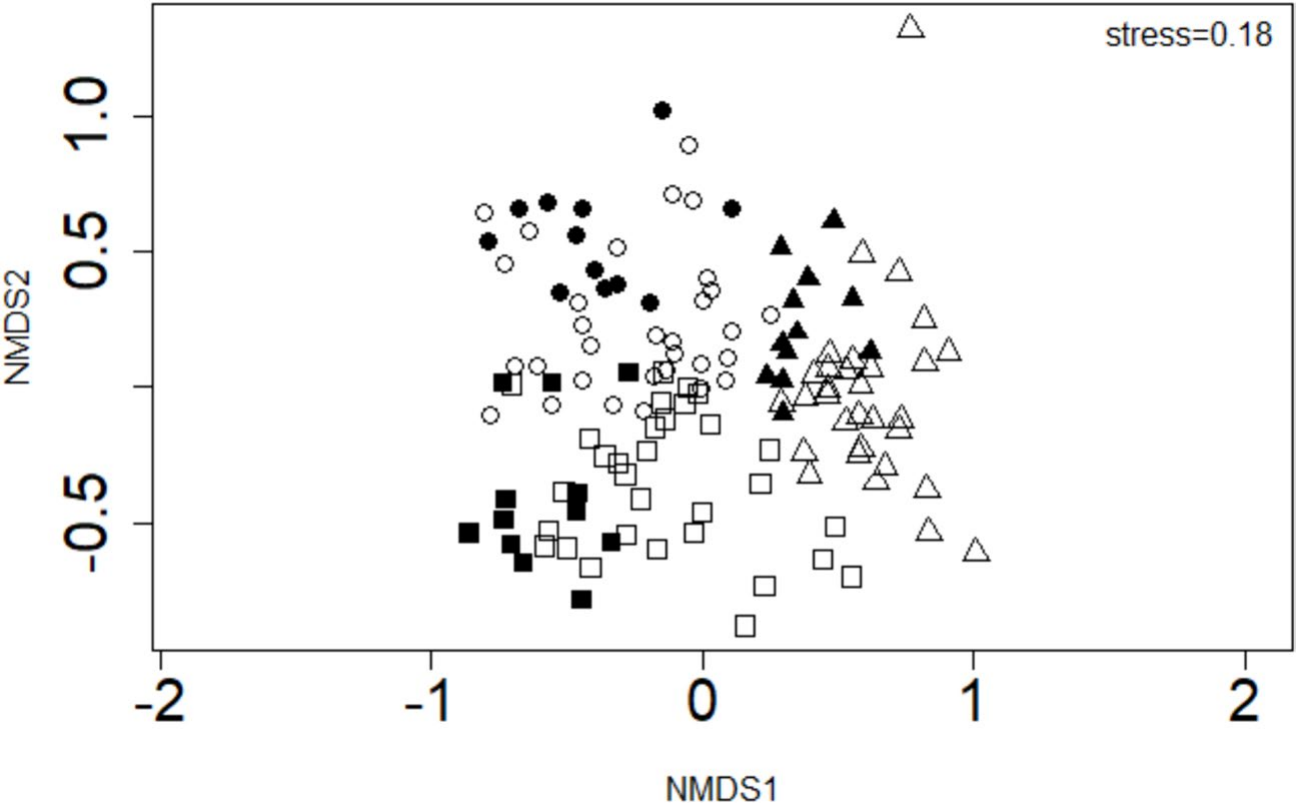
Non-metric multidimensional scaling for metal levels observed in the liver (□ Inanda Dam; ■ Nagle Dam), gill (∆ Inanda Dam; ▲ Nagle Dam), and muscle (○ Inanda Dam; ● Nagle Dam) of *Coptodon rendalli*

Metal levels observed in the liver, gills, and muscle of *C. rendalli* are presented in Fig. 2. A significant difference was observed for Al (w = 267, p < 0.05), Cr (w = 68, p < 0.05), and Mn (w = 71.50, p < 0.05) levels in the gill between the Inanda and Nagle dam populations (Fig. 2). Liver showed significant differences for Cd (w = 104, p < 0.05), Cr (w = 76, p < 0.05), Fe (w = 36, p < 0.05), Mo (w = 77, p < 0.05), and Zn (w = 335, p < 0.05) whereas muscle exhibited significant difference for Al (w = 330, p < 0.05), Cd (w = 268, p < 0.05), Cr (w = 95.7, p < 0.05), Fe (w = 20, p < 0.05), Mo (w = 55.5, p < 0.05) and Pb (w = 283, p < 0.05) (Fig. 2). The Al, Sb, Cd, Mn, and Zn were generally higher at Inanda Dam for all tissues whereas Cr, Fe, Mo, and Pb were higher at Nagle Dam (Fig. 2). Aluminium, Pb, and Zn distribution exhibited a descending order liver > gill > muscle whereas Sb, Cr, and Mn exhibited gill > liver > muscle at both dams (Fig. 2). Molebdinum showed a descending order muscle > gill > liver at both dams whereas Cd showed Muscle > liver > gill at Inanda Dam and liver > gill > muscle at Nagle Dam. Moreover, Fe showed a descending order gill > liver > muscle at Inanda Dam and liver > gill > muscle at Nagle Dam (Fig. 2). Corroborating these trends, non-metric multidimensional scaling showed a clear separation for metal levels in the liver, gill, and muscle between the two dams (MANOVA, p < 0.001), and a similar trend was observed for organs in each dam (MANOVA, p < 0.001) (Fig. 3). The three organs showed no significant difference in dispersion between the two dams (DISPER, p > 0.05).

### Correlation between fish length and metal, and between metals

Levels of the two populations were pooled for correlation tests and no relationship was observed for fish length metals in the gill. However, strong and moderate inter-metal relationships were observed for a few metals. The only strong relationships observed in the gills were Sb-Fe (*ρ* = 0.76, p < 0.05) and Sb-Mn (*ρ* = 0.62, p < 0.05) whereas moderate relationships were observed for Al-Sb (*ρ* = 0.51, p < 0.05), Al-Cd (*ρ* = -0.44, p < 0.05), Sb-Cd (*ρ* = -0.47, p < 0.05), Al–Fe (*ρ* = 0.43, p < 0.05) and Fe–Mn (*ρ* = 0.54, p < 0.05). Similarly, liver showed a strong relationship for Cr–Mo (*ρ* = 0.71, p < 0.05) with moderate relationships being observed for Al-Cr (*ρ* = -0.41, p < 0.05), Al–Fe (*ρ* = -0.52, p < 0.05), Cd-Fe (*ρ* = 0.41, p < 0.05), Al-Mo (*ρ* = -0.43, p < 0.05) and Fe-Mo (*ρ* = 0.53, p < 0.05). The muscle showed a strong relationship for Cr–Mo (*ρ* = 0.74, p < 0.05) and moderate relationships for Fe-Zn (*ρ* = 0.45, p < 0.05) and Mo-Zn (*ρ* = 0.50, p < 0.05).

### Human health risk assessment

**Fig. 4:**
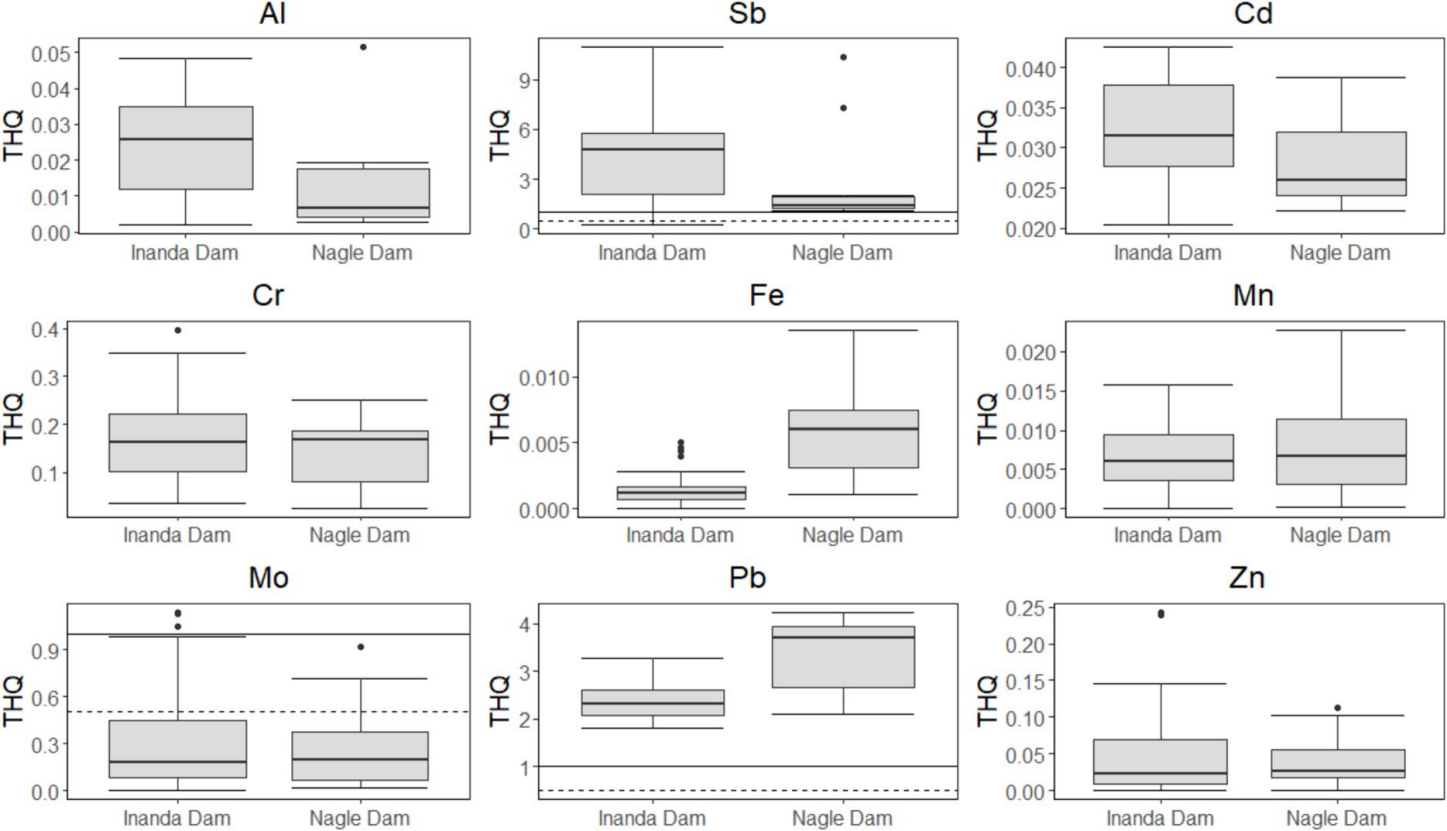
The target hazard quotient observed in the muscle of *Coptodon rendalli* sampled from the Inanda and Nagle dams. The solid and dashed lines in each plot indicate the thresholds of 1 and 0.5, respectively

**Fig. 5:**
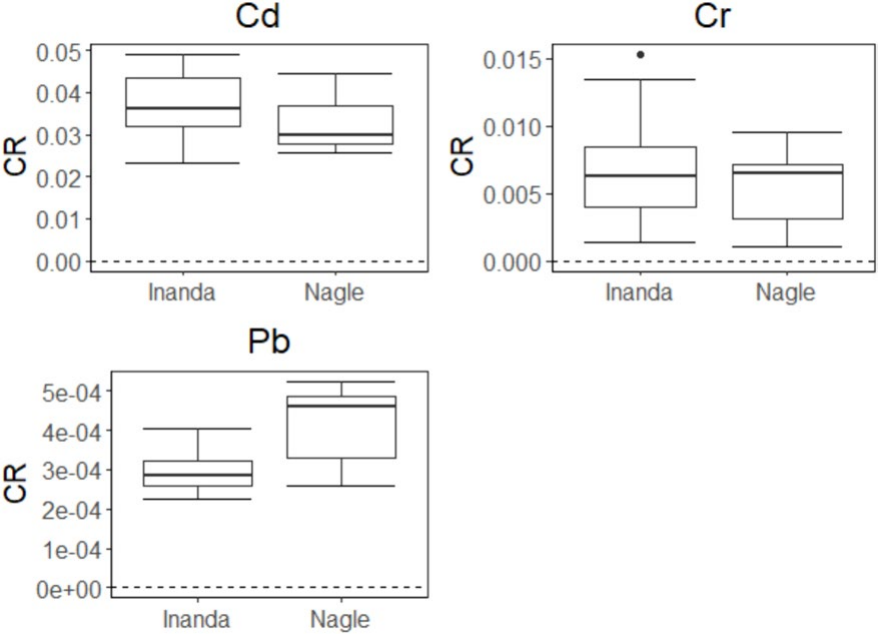
The carcinogenic risk quotient observed for three metals in the muscle of *Coptodon rendalli* from Inanda and Nagle dams

The non-carcinogenic THQs observed for the muscle of *C. rendalli* are presented in Fig. 4. Antimony, Mo, and Pb exhibited THQs exceeding the threshold of 1 at both dams. Moreover, Mo showed THQs of > 1 for 10% of its population with approximately 25% exhibiting the quotient > 0.05 for the Inanda Dam population (Fig. 4). The HI was greater than 1 for all metals at both dams (Table 2). Moreover, the carcinogenic risk for Cd, Cr, and Pb concentrations exceeded the standard value of 10^–6^ at both dams (Fig. 5).

**Table 2:** The hazard index observed in the muscle of Coptodon rendalli sampled from the Inanda and Nagle dams

Dams	Al	Sb	Cd	Cr	Fe	Mn	Mo	Pb	Zn
Inanda Dam	0.74	141.62	0.99	5.35	0.05	0.21	10.33	74.20	1.61
Nagle Dam	0.16	31.77	0.33	1.73	0.07	0.10	3.39	40.51	0.49

## Discussion

### The overall fish health

The overall fish health showed no difference between the two dams, with fish sizes and growth showing similar patterns. The *b* value ranged from 1.174 to 2.066, hence, a negative allometric growth (b < 3) at both dams. These results are similar to those observed in other related studies across the world (Jisr et al., 2018; Misra et al., 2024; Radwan et al., 2022). The length–weight relationship may differ with species due to their inherited body shape and other physiological factors such as maturity and spawning; however, the *b* value ranging from 2.5 to 4 is considered ideal (Arafat & Bakhtiyar, 2022; Froese, 2006). In the present study, the *b* values were below the ideal range for freshwater fish, suggesting that fish from both dams were thinner for their length (Jisr et al., 2018). Factors such as sex, season, stress, water quality, and food availability could influence the *b* value resulting in negative allometric conditions for fish populations (Dinh et al., 2022; Mommsen et al., 2001). Dang and Wang (2012) reported negative allometric growth in a fish population exposed to mercury whereas Whiterod et al. (2018) reported negative allometric growth due to flow alterations and thermal pollution. Given that the Inanda Dam receives water from the polluted uMsunzud River, pollution as a potential driver for the observed *b* may not be dismissed whereas other factors may be implicated for the Nagle Dam population.

Moreover, the condition factor may serve as an indicator of the physiological state of fish concerning its welfare (Famoofo & Abdul, 2020). In the present study, the condition factor ranged from 1.53 – 3.86 at Nagle Dam and 1.73 – 3.11 at Inanda Dam. These findings were comparable to those reported by Bagenal and Tesch (1978) (2.9 – 4.8) for freshwater fish, and higher than those reported by Famoofo and Abdul (2020) and Jisr et al. (2018). However, Ajibare et al. (2020) emphasised that a value close to, or above 1 is more desirable while a value below 0.80 indicates extremely poor health. The condition factor observed in the present study has shown no difference between the two dams and they were above Ajibare et al. (2020) critical value of 1. The condition factor may vary with species and locations as they reflect the feeding intensity, physiological activities, environmental quality, and growth rates in fish (Uneke, 2017). Although the condition factor is reported to be a reliable indicator of the well-being of fish populations in fisheries (Uddin & Ghosh, 2021) and environmental studies (Cavraro et al., 2019), it was not conclusive in discriminating the two populations in the present study.

Similarly, the hepatosomatic index (HSI) has shown no difference between the two dams, and the values observed were within the normal range as per Munshi and Dutta (1996). These values were comparable to those observed on fish exposed to Cd (Idrus et al., 2022) and metal-polluted Karasu River (Dane & Şişman, 2020). The hepatosomatic index may change due to starvation as it is related with liver energy reserves and metabolic activities (Yang et al., 2019). Moreover, HSI may increase as a response to enhanced detoxification capacity (Tenji et al., 2020). In the present study, the HSI has not deviated from the normal range at the two dams.

### Metal level in fish tissues

Metal pollution of aquatic environments has been a cause for concern since metals can persist in the environment for a long time and have the ability to accumulate in the tissues of aquatic organisms (Aziz et al., 2023). However, factors such as pH, temperature, salinity, and the presence of other chemicals can influence metal bioavailability for bioaccumulation to take place (Martínez-Durazo et al., 2021). Moreover, bioaccumulation is dependent on the organism’s lifespan, position on trophic level, level of metals exposed to, and the metabolic capacity and size of fish (Okati et al., 2021; Rajeshkumar & Li, 2018). Nevertheless, the organ roles in organisms have also been found to influence bioaccumulation and distribution within the body. In the present study, most metals showed higher levels in the liver, followed by the gill and muscle at both dams. This trend was comparable to what was reported in other related studies in freshwater ecosystems (Marr et al., 2017; Maurya & Malik, 2018; Pan et al., 2022).

However, other metals showed different trends in the present study. Antimony, Fe, and Mo showed gill > liver > muscle pattern at both dams. The gill > liver > muscle is the second popular trend as both liver and gills are playing roles in detoxification and osmoregulation, respectively (Roberts, 2012; Wang & Wang, 2016). Bashir et al. (2012) observed the gill > liver > muscle trend for Al, Zn, and Pb in *Arius thalassinus* and liver > gill > muscle for As, Fe, Cd, and Zn in *Pennahia anea*. Moreover, Bawuro et al. (2018) observed the liver > gill > muscle trend for Cu and Zn and gill > liver > muscle for Pb and Cd in Tilapia species. Unpopular trends of muscle > gill > liver and muscle > gill > liver have also been reported in various studies for various metals such as Cd, Sr, Se, Mn, and Sb (Arantes et al., 2016; Marr et al., 2017; Shah et al., 2021; Zaghloul et al., 2024). It is evident that metals have different affinity towards organs and species, hence, different trends have been reported for different species globally. The exposure route has also been reported to be among the factors influencing metal distribution in fish (Wang & Wang, 2016).

The liver is the primary site for biotransformation and detoxification, and most metals reaching the liver are those coming through diet (Roberts, 2012). Some metals can exhibit high levels and still induce no toxicity as they are essential for the physiological processes of organisms (Hashim et al., 2014). In the present study, the liver of *C. rendalli* exhibited Al > Fe > Zn > Cr > Mo > Cd > Pb > Sb > Mn and Al > Fe > Zn > Cr > Mo > Pb > Sb > Mn > Cd trends at the Inanda and Nagle dams, respectively. Metals such as Fe and Zn are essential for physiological processes whereas Al is toxic at high levels (Rajeshkumar & Li, 2018). These three metals also occur at high levels in natural environments without causing adverse effects on aquatic biota. In contrast, toxic metals occur at low levels in natural environments, so their minute levels in the liver are not peculiar. Nevertheless, these very low levels may be toxic to organisms, particularly when they occur in combination with other metals. Therefore, any level of these heavy metals in animal tissues is concerning. These trends are not unique as they concur with those observed in other related studies (Bawuro et al., 2018; El-Moselhy et al., 2014; Pan et al., 2022). The role of organs and metal affinity plays a huge role in metal distribution in organisms (Jayaprakash et al., 2015), which could explain the observed variability of metal level trends in the liver.

Gills play a role in osmoregulation due to their immediate contact with the external water environment and respiratory function in fish (Jayaprakash et al., 2015; Njinga et al., 2023). Similar to the liver, the Al, Fe, and Zn levels were relatively higher compared to the others at both dams. Another metal that has proven some affinity to gill tissue is Mn (Crafford & Avenant-Oldewage, 2011; Niemiec & Wisniowska-Kielian, 2015). However, no clear trend relative to other metals was observed for Mn in the present study. Moreover, levels for most metals in the gills were comparable to those reported in other related studies (Bashir et al., 2012; Bawuro et al., 2018; Zaghloul et al., 2024).

Muscle has also shown a similar trend with Al, Fe, and Zn exhibiting higher levels compared to other metals. Egbe et al. (2023) reported Fe > Mn > Cr > Cu > Pb > Zn > Cd > As trend for *Coptodon kottae* and Fe > Mn >  > Pb > Cr > As > Zn > Cd for *Oreochromis niloticus*. Moreover, El-Moselhy et al. (2014) reported higher Fe and Zn levels compared to Cd, Mn, and Pb in the muscle of numerous fish species. In contrast, Marr et al. (2017) found Ba and Mo levels to be higher than those of Al, Fe, and Zn in the muscle of *Labeo rosae* from metal-contaminated water bodies*.* However, levels in the muscle were substantially lower compared to those observed in the liver and gills. This was expected as there are no metal metabolic activities in the muscle, hence, it is not a target organ (Jezierska and Witeska, 2006; Bakhshalizadeh et al., 2022). However, the metal levels observed in the muscle of *C. rendalli* were comparable to those observed in fish from contaminated water bodies (Njinga et al., 2023; Pan et al., 2022; Rajeshkumar & Li, 2018).

### Comparison between dams

The Inanda Dam receives water from a highly polluted uMsunduzi River which is draining a highly industrialized catchment (Adeyinka et al., 2018; Misra et al., 2024). Nevertheless, no definite trend was observed for metal concentrations between the two dams. The Al, Sb, Cd, Mn, and Zn were generally higher at Inanda Dam whereas Cr, Fe, Mo, and Pb were higher at Nagle Dam for all tissues. Activities such as mining, power generation plants, metallurgic industries, wastewater works and urbanization may drive metal pollution in river systems (González-Fernández et al., 2018), which could be the explanation for the increased concentrations in the Inanda Dam. However, the river stretch and catchment feeding the Nagle Dam is characterised by negligible activities such as subsistence farming, and game farms and livestock roaming. Moreover, the dam is bordered by mountains which give the bottom substrate unique characteristics. According to Ali et al. (2019), metal concentration increase in freshwater systems may also be influenced by natural sources such as rock weathering, erosion and atmospheric depositions. Given that the Nagle Dam is located approximately 40 km upstream of industrial activities threatening Inanda Dam, and it is bordered by mountains, the atmospheric deposition and the geological characteristics may be the explanations for metal concentrations at Nagle Dam.

### Correlation between fish length and metal, and between metals

No fish length-metal relationships were observed in all the three tissues*.* However, Jiang et al. (2022) reported that metal levels may decrease with increasing fish length. Corroborating findings of the present study, Hashim et al. (2014) reported weak to no relationship between fish size and Cd and Pb concentrations whereas Marr et al. (2017) reported no relationship between fish length and metals in the liver, gill, and muscle of *L. rosae*. In contrast, Balzani et al. (2022) observed a strong positive relationship between fish length and Cu and Zn levels in *Pseudorasbora parva* and *Alburnus alburnus*, respectively. No definite relationships between fish length and metal levels in different tissues were observed in the present study. This could probably be associated with the fact that metals behave differently when they are mixed with other chemicals and their affinity differs with tissues and species (Niemiec & Wisniowska-Kielian, 2015).

Moreover, no definite trend was observed for inter-metal relationships across all tissues. Some metals may have synergistic whereas others could have antagonistic interactions in animal tissues. Rajeshkumar and Li (2018) reported a negative moderate relationship for Cd–Pb in the gill, a strong positive relationship for Cd–Pb, and a moderate positive relationship for Cr-Pb in the liver of *Cyprinus carpio*. Similarly, Afandi et al. (2018) reported opposite relationships between metals for muscle and liver tissues in pelagic fish species. Another study observed no definite relationship between metals during the summer and winter seasons (Ali et al., 2020a, 2020b). It is evident that the inter-metal relationship could be driven by various factors and their interpretations should be carefully thought out. Different species showed different associations for the same metals and similar patterns were observed in different media such as water and sediment (Engdaw et al., 2022; Jin et al., 2022).

### Non-carcinogenic human health risk assessment

Fish consumption is highly recommended due to high-quality proteins, vitamins, long-chain polyunsaturated fatty acids (PUFAs), and eicosapentaenoic acid/docosahexaenoic acid (Barone et al., 2021). However, contamination of water bodies is threatening these potential benefits as fish consumption is regarded as one of the most crucial pathways of human exposure to heavy metals (Ahmed et al., 2016). Studies have shown that consuming fish from polluted water body poses health risks to consumers (Adegbola et al., 2021; Kumari & Kumar Maiti, 2019; Lebepe et al., 2020). This was also observed in the present study with Sb, Mo, and Pb in the muscle of *C. rendalli* from the Inanda and Nagle dams showing levels exceeding acceptable levels for human consumption. Moreover, Cr level was significant with the THQs just below 0.5, which suggests that it has to be monitored closely. Other metals showed THQs of < 1 which implies that they are safe levels for human consumption. However, the calculations were made based on the assumption that a 70 kg adult consumes a 150 g portion once a week, therefore, a change in one of these parameters may change the risk quotient.

For example, consumption by children may result in higher risk compared to what was observed in the present study. Therefore, the THQs observed in this study remain a cause for concern, particularly those that showed quotients ranging from 0.4 to 0.6. Moreover, given that metals fixed in sediment may be re-suspended back into the water column due to changes in physico-chemical parameters such as pH and others, regular monitoring of metal levels in fish muscle should be implemented to ensure that any deviation from the current state may be detected in time. These dams are bordered by poor rural communities which practice artisanal fishing. Moreover, the Inanda Dam is home for angling competitions and some participants are taking fishes with them for consumption which expose them to uncertainties with regard to their health. Therefore, in this region, consuming *C. rendalli* as the primary source of fish should be done with caution regarding the quantity consumed. Further studies are required in these two water bodies as local communities rely on fish living there as a crucial source of protein.

### Carcinogenic human health risk assessment

Carcinogenic risk was also assessed to determine the probability of developing over a lifetime of an individual when exposed to a potential carcinogen (Adegbola et al., 2021). Three metals were assessed for cancer risk as there is no slope factor for other metals in the literature and they all exceeded the standard risk quotient of 10^–6^. The Cr and Cd exhibited the CR of > 10^–3^ and > 10^–2^ at both the Inanda and Nagle dams. These risk quotients were comparable to those observed by Debipersadh et al. (2023) and higher than those reported by (Huang et al., 2019) in metal-polluted water bodies. Lead showed a CR of > 10^–4^ at both dams. The Pb risk quotient was lower than those reported by Adegbola et al. (2021) for *Clarias gariepinus* and *Sarotherodon melanotheron* and higher than those reported by Hossain et al. (2019) on *Nandus nandus*, *Glossogobius giuris*, *Macrognathus pancalus* and *Lepidocephalichthys guntea* in water bodies impacted by industrial activities. No differences were observed for Cd, Cr, and Pb in terms of risk factors between the two dams. It is evident that Cd, Cr, and Pb concentrations observed in the muscle of *C. rendalli* are a cause for concern as they have the potential to cause cancer to fish consumers in the long run.

## Conclusion

Metal distribution exhibited popular trends of liver > gill > muscle and gill > liver > muscle for most metals with Al, Fe, and Zn showing substantially higher levels compared to others. Moreover, Al, Sb, Cd, Mn, and Zn were generally higher at Inanda Dam for all tissues whereas Cr, Fe, Mo, and Pb were higher at Nagle Dam. The overall fish health was not severely impacted by the pollution, however, the level of metals in these water bodies is becoming a cause for concern. Moreover, Sb, Mo, and Pb exceeded the acceptable threshold for human consumption whereas Cr showed a significant value. These findings suggest that consuming *C. rendalli* from the uMgeni River system such that an adult weighing 70 kg eats 150 g portion once a week could result in adverse health effects. Moreover, Cd, Cr and Pb showed CR of > 10^–6^ suggesting a potential for cancer risk over time. These results provide a warning to local and international communities residing next to water bodies impacted by metallurgic pollution about the possible health risks of consuming metal-polluted fish. The limitation of the study is that metal concentrations in the water and sediment were not analysed which would have helped reflect on the dynamics of metals between the three matrices. Fish advisories are recommended in this catchment to sensitize local communities on the health risks associated with consuming *C. rendalli* from this river system. Additionally, more related studies are recommended in water bodies impacted by various anthropogenic activities, particularly, those flowing through poor communities to ensure the safety of fish consumers.

## Supplementary Information

Below is the link to the electronic supplementary material.

Supplementary file1 (DOCX 117 KB)

## Data Availability

Data is available upon request.

## Declarations

All authors have read, understood, and complied as applicable with the statement on “Ethical responsibilities of Authors” as found in the Instructions for Authors.
