# Supplementary material for: Metal distribution in three organs and edibility assessment on Coptodon rendalli from the Umgeni River impacted by metallurgic industrial activities
Source: Environ Monit Assess. 2024 Jul 17;196(8):742. doi: 10.1007/s10661-024-12875-w (PMC11519209; doi:10.1007/s10661-024-12875-w)
Supplement: Supplementary file 1 — Supplementary file1 (DOCX 117 KB) [file 10661_2024_12875_MOESM1_ESM.docx]

**Appendix**

**Appendix 1.** Morphometrics and fish indices observed for *Coptodon rendalli* from the Inanda and Nagle dams

| Dams | Total length (cm) | Standard length (cm) | Fish weight (g) | Condition factor | Hepatosomatic index |
| --- | --- | --- | --- | --- | --- |
| Inanda Dam | 26.42 | 20.40 | 435.00 | 2.65 | 1.53 |
| Inanda Dam | 26.42 | 20.40 | 380.00 | 2.32 | 0.52 |
| Inanda Dam | 26.92 | 30.80 | 656.00 | 1.43 | 1.68 |
| Inanda Dam | 24.13 | 19.80 | 325.00 | 2.13 | 1.32 |
| Inanda Dam | 29.21 | 38.70 | 680.00 | 1.61 | 1.66 |
| Inanda Dam | 26.67 | 20.50 | 545.00 | 3.29 | 1.29 |
| Inanda Dam | 26.67 | 20.50 | 400.00 | 2.41 | 1.06 |
| Inanda Dam | 26.67 | 19.50 | 485.00 | 3.30 | 0.90 |
| Inanda Dam | 24.13 | 21.00 | 380.00 | 2.16 | 1.04 |
| Inanda Dam | 27.94 | 19.85 | 560.00 | 3.65 | 1.31 |
| Inanda Dam | 24.89 | 19.85 | 420.00 | 2.74 | 0.47 |
| Inanda Dam | 30.23 | 21.59 | 670.00 | 4.00 | 0.56 |
| Inanda Dam | 28.70 | 21.59 | 670.00 | 2.13 | 1.11 |
| Inanda Dam | 26.16 | 18.50 | 398.00 | 3.27 | 1.34 |
| Inanda Dam | 25.40 | 20.00 | 375.00 | 4.11 | 0.97 |
| Inanda Dam | 26.92 | 30.60 | 665.00 | 1.47 | 1.11 |
| Inanda Dam | 26.42 | 20.40 | 465.00 | 2.84 | 1.14 |
| Inanda Dam | 26.67 | 20.50 | 490.00 | 2.96 | 0.97 |
| Inanda Dam | 24.13 | 19.50 | 340.00 | 2.31 | 0.95 |
| Inanda Dam | 25.40 | 18.20 | 390.00 | 3.12 | 1.39 |
| Inanda Dam | 19.05 | 11.00 | 150.00 | 3.66 | 0.90 |
| Inanda Dam | 41.00 | 35.10 | 1475.00 | 2.29 | 0.66 |
| Inanda Dam | 36.50 | 31.10 | 1425.00 | 3.03 | 1.02 |
| Inanda Dam | 30.40 | 24.60 | 675.00 | 2.60 | 1.20 |
| Inanda Dam | 36.50 | 30.70 | 1110.00 | 2.44 | 1.34 |
| Inanda Dam | 24.90 | 21.50 | 355.00 | 1.91 | 2.20 |
| Inanda Dam | 24.00 | 20.40 | 330.00 | 2.01 | 1.70 |
| Inanda Dam | 37.50 | 32.50 | 1230.00 | 2.33 | 0.71 |
| Inanda Dam | 38.20 | 24.00 | 505.00 | 2.07 | 1.05 |
| Inanda Dam | 24.90 | 23.00 | 640.00 | 2.92 | 1.45 |
| Inanda Dam | 23.10 | 23.10 | 608.00 | 2.74 | 2.02 |
| Nagle Dam | 28.19 | 24.13 | 120.00 | 1.67 | 3.23 |
| Nagle Dam | 27.43 | 22.35 | 520.00 | 3.50 | 1.08 |
| Nagle Dam | 30.99 | 24.89 | 825.00 | 3.86 | 1.34 |
| Nagle Dam | 38.61 | 32.51 | 1210.00 | 3.52 | 0.58 |
| Nagle Dam | 38.50 | 34.10 | 400.00 | 1.97 | 1.33 |
| Nagle Dam | 34.50 | 29.30 | 600.00 | 2.39 | 0.98 |
| Nagle Dam | 29.80 | 25.70 | 495.00 | 2.92 | 0.51 |
| Nagle Dam | 39.30 | 32.60 | 520.00 | 1.53 | 0.65 |
| Nagle Dam | 29.90 | 24.60 | 385.00 | 2.59 | 0.81 |
| Nagle Dam | 34.20 | 28.50 | 720.00 | 3.11 | 1.06 |
| Nagle Dam | 32.80 | 28.50 | 585.00 | 2.53 | 1.04 |
| Nagle Dam | 38.00 | 31.20 | 850.00 | 2.80 | 0.53 |


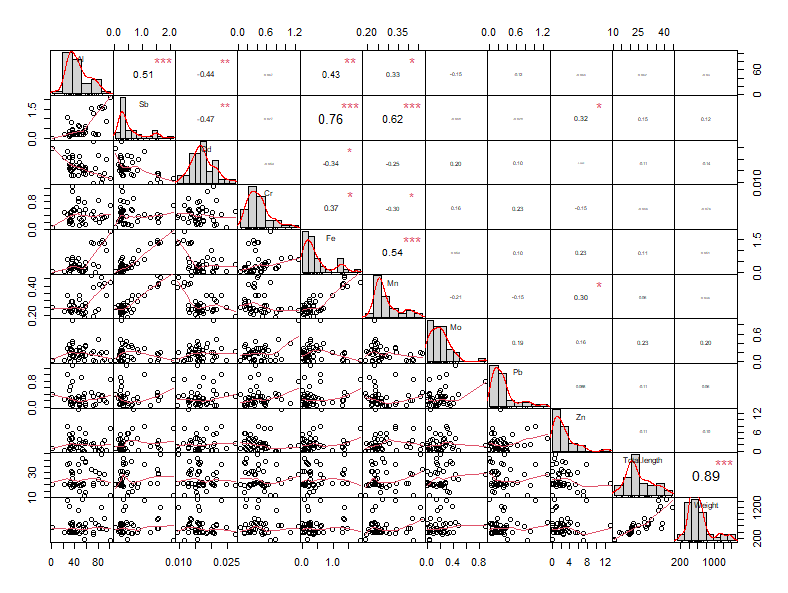


**Appendix 2.** Inter-metal and metal-fish sizes correlation coefficients observed in *Coptodon rendalli* from the Inanda and Nagle dams


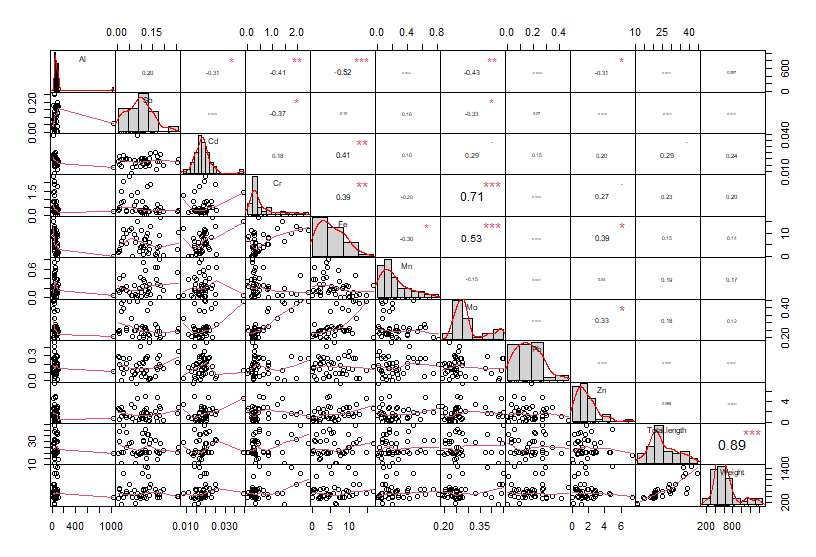


**Appendix 3.** Inter-metal and metal-fish sizes correlation coefficients observed in the liver *Coptodon rendalli* from the Inanda and Nagle dams


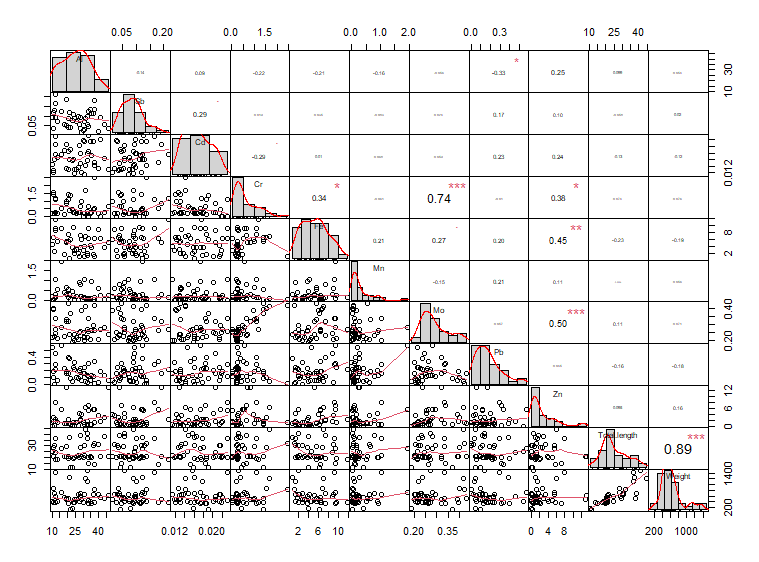


**Appendix 4.** Inter-metal and metal-fish sizes correlation coefficients observed in *Coptodon rendalli* from the Inanda and Nagle dams
